# A Prospective Investigation of Bispecific CD19/22 CAR T Cell Therapy in Patients With Relapsed or Refractory B Cell Non-Hodgkin Lymphoma

**DOI:** 10.3389/fonc.2021.664421

**Published:** 2021-05-25

**Authors:** Ying Zhang, Jiaqi Li, Xiaoyan Lou, Xiaochen Chen, Zhou Yu, Liqing Kang, Jia Chen, Jin Zhou, Xiangping Zong, Zhen Yang, Minghao Li, Nan Xu, Sixun Jia, Hongzhi Geng, Guanghua Chen, Haiping Dai, Xiaowen Tang, Lei Yu, Depei Wu, Caixia Li

**Affiliations:** ^1^ Jiangsu Institute of Hematology, The First Affiliated Hospital of Soochow University, Suzhou, China; ^2^ National Clinical Research Center for Hematologic Diseases, Jiangsu Institute of Hematology, The First Affiliated Hospital of Soochow University, Suzhou, China; ^3^ Institute of Blood and Marrow Transplantation, Collaborative Innovation Center of Hematology, Soochow University, Suzhou, China; ^4^ Shanghai Unicar-Therapy Bio-medicine Technology Co., Ltd, Shanghai, China; ^5^ Institute of Biomedical Engineering and Technology, Shanghai Engineering Research Center of Molecular Therapeutics and New Drug Development, School of Chemistry and Molecular Engineering, East China Normal University, Shanghai, China

**Keywords:** bispecific chimeric antigen receptor, CD19/22, relapsed or refractory, B cell non-Hodgkin lymphoma, cellular kinetics

## Abstract

**Background:**

The use of T cells expressing chimeric antigen receptor (CAR T) engineered to target CD19 constitutes breakthrough treatment for relapsed or refractory B cell non-Hodgkin lymphoma (R/R B-NHL). Despite improved outcomes, high relapse rate remains a challenge to overcome. Here, we report the clinical results and the pharmacokinetics of bispecific CD19/22 CAR T in patients with R/R B-NHL.

**Methods:**

We performed a prospective, single-arm study of bispecific CD19/22 CAR T cells in R/R B-NHL. We analyzed the safety and efficacy and investigated the kinetic profiles of the CAR T cells. CAR transgene levels were measured using quantitative polymerase chain reaction, and correlation analyses of pharmacodynamic markers and product characteristics, disease conditions, clinical efficacy and adverse events were performed.

**Results:**

From August 2017 to September 2020, a total of 32 patients with CD19/22 CAR T administration were analyzed. The overall response rate was 79.3%, and the complete response rate was 34.5%. The progression-free survival (PFS) and overall survival (OS) rates at 12 months were 40.0% and 63.3%, respectively. Among patients who had a CR at 3 months, the PFS and OS rates at 12 months were 66.7% and 100%, respectively. Severe cytokine release syndrome (sCRS) (grade 3 and higher) occurred in nine patients (28.1%). Grade 3 or higher neurologic events occurred in four patients (12.5%). One patient died from irreversible severe CRS-associated acute kidney injury. Long-term CAR T cells persistence correlated with clinical efficacy (133 days vs 22 days, P = 0.004). Patients treated with more than three prior therapies and presenting extranodal organ involvement had lower maximal concentration (C_max_) values than other patients. Responders had higher C_max_ and area under the curve values than non-responders. Tumour burden and C_max_ were potentially associated with the severity of CRS.

**Conclusions:**

This study demonstrates the safety and potential clinical efficacy of bispecific CD19/22 CAR T cells in patients with R/R B-NHL and highlights the importance of measuring kinetic parameters in PB to predict efficacy and safety in clinical applications of CAR T cell therapy.

**Clinical Trial Registration:**

https://www.clinicaltrials.gov/ct2/show/NCT03196830, identifier NCT03196830.

## Introduction

Chimeric antigen receptor T (CAR T) cells are genetically engineered to allow T cells to recognize and interact with tumor cells, leading to target cell lysis and the subsequent effective and profound clearance of tumor cells (1). Currently, CD19-targeting CAR T cells are the most widely used and exhibit remarkable efficacy in patients with relapsed or refractory acute lymphoblastic leukaemia (R/R ALL) and relapsed or refractory B cell non-Hodgkin lymphoma (R/R B-NHL), with a complete response (CR) rate of 82% to 93% (2–4) and 40% to 53% (5–7), respectively. Despite this impressive efficacy, the benefits are often transient, and relapse occurs in 30%–50% patients who receive CD19 CAR T cells infusion (8, 9). The main reasons for relapse might be tumour antigen loss and a lack of CAR T cells persistence (10). Recently, multiple studies have shown that bispecific CAR T cells or the sequential use of different target CAR T strategies may prolong the persistence of CAR T cells and may overcome CD19-negative relapse (8, 11, 12). Zhou and colleagues previously reported that the sequential infusion of CD19 and CD22 CAR T cells was a feasible and safe clinical treatment for B cell malignancies (13). Other reports also showed that an infusion of bispecific CAR T cells or sequential infusion of CAR T cells with different targets was safe and efficacious and may have reduced the relapse rate caused by antigen escape in B cell malignancies (14–16).

Unlike the pharmacokinetics of conventional drugs whose levels decrease over time, CAR T cells are “living drugs” that undergo a rapid proliferative phase *via* the antigen-specific activation of 4-1BB/CD28-CD3zeta signaling, which may reach up to 1,000-fold expansion (17, 18). This robust proliferative phase may offset or even counteract the effects of the elimination phase, which significantly increases the maximal blood concentration and prolongs the persistence of CAR T cells *in vivo*. Additionally, most CAR T cells undergo programmed death after tumor eradication (19, 20) but a few CAR T cells may transform into memory cells with longer lifespans and much slower elimination rates, leading to long-term immunological surveillance (21). Some reports even revealed a second increase in the peripheral blood (PB) concentration of CAR T cells several months after CAR T cells administration in response to antigen reappearance due to tumor relapse (22). All these reports reveal the unique pharmacokinetic features of CAR T cells that differ from conventional drugs. Thus, characterization of the cellular kinetics of CAR T cells and determination of kinetic-related factors are important for understanding and predicting the efficacy and toxicities of CAR T cells.

Some published reports have documented CD19 CAR T pharmacology in patients with B cell ALL and chronic lymphoblastic leukaemia (CLL) (17, 23), helping people understand the correlations between the characterization of CD19 CAR T cells kinetics and the safety and effectiveness of these therapies. Currently, only a few scattered papers have reported changes in the cellular kinetics of CD19 CAR T cells in adults with NHL (9, 24), and no article has reported the pharmacokinetics of dual-target CAR T cells; thus, the cellular kinetics of CAR T cells after an infusion in patients with lymphoma remain poorly understood. Moreover, while the effects of a therapy targeting a single antigen have been clarified, the effect of bispecific CAR T cells targeting both CD19 and CD22, which present different behaviours in expansion and persistence, are not clear. Additionally, the structure, product characteristics and infusion dose of CAR T cells in the published papers are not consistent, which may indicate different kinetic behaviors.

Here, we report the clinical safety and efficacy of CAR T cells in 32 patients with R/R B-NHL treated with 2nd generation 41BB-CD3zeta CAR T cells bispecific for CD19/22. For the first time, we comprehensively summarize the cellular kinetics of bispecific CD19/22 CAR T cells.

## Methods

### Study Design and Patients

Data were collected from a single-arm study of bispecific CAR T cell therapy targeting CD19/22 in patients with R/R B-NHL (NCT03196830). The study was approved by the Institutional Ethics Committee of the First Affiliated Hospital of Suzhou University and completed through a collaboration with Shanghai Unicar-Therapy Bio-medicine Technology Co., Ltd. All patients provided written informed consent in accordance with the Declaration of Helsinki. The detailed inclusion and exclusion criteria are available in the **Supplementary Materials**.

### Study Procedures and Treatment

Patients were enrolled following the screening and confirmation of eligibility and then underwent leukapheresis for the isolation of peripheral blood mononuclear cells (PBMCs) to manufacture the CD19/22 CAR T cells. The final CD19/22 CAR T cell products were washed, cryopreserved, and tested for identity, potency, sterility, and adventitious agents. After meeting the acceptance criteria, the products were shipped back to the clinical sites.

Low-dose preconditioning consisting of fludarabine (at a dose of 30 mg per square metre of body-surface area per day) and cyclophosphamide (at a dose of 300 mg per square meter per day) were accomplished on days -5, -4, and -3. CD19/22 CAR T cells fractionated infusion were split over 3 days (day 0, 10%; day 1, 30%; day 2, 60%) at a total dose of 3.690 ∗ 10^8^ to 3.285 ∗ 10^9^ CAR T cells (median dose of 8.258 ∗ 10^8^ CAR T cells).

The response was the primary objective and was assessed using positron emission tomography-computed tomography (PET-CT) or control CT at months 1, 3, 6, 9, 12, 18, 24 and 36. The disease response and duration of response (DOR) were determined according to the “2014 Lugano Classification” (25). Adverse events (AEs) were detected beginning with the CD19/CD22 CAR T cells infusion. AEs were graded according to the NCI Common Terminology Criteria for Adverse Events (CTCAE), version 5.0. Cytokine release syndrome (CRS) and neurological events, which were also called immune effector cell-associated neurotoxicity syndrome (ICANS), were defined, graded and managed according to the American Society for Blood and Marrow Transplantation (ASBMT) consensus (26). Cellular kinetics was the secondary objective of this trial. Only data from patients who successfully received CAR T cells infusions were pooled in the study.

### Generation of CD19/22 CAR T Cells

T cells from leukapheresis products were isolated using anti-CD3 magnetic beads (Miltenyi Biotec, Bergisch-Gladbach, Germany). T cells were then stimulated with monoclonal anti-CD3/CD28 antibodies (Miltenyi Biotec, Bergisch-Gladbach, Germany). Two days after the initial T cells activation step, the T cells were transduced with a lentivirus encoding the CD19/22-4-1BB-CD3 ζ transgene. Cells were cultured in AIM-V media (Gibco, NY, USA) supplemented with 10% autologous serum, 100 IU/ml IL-2, 5 ng/ml IL-7, and 5 ng/ml IL-15 for 12 days. Quality checks were performed during the CAR T cells manufacturing process. The transduction efficiency, percentages of CD3^+^ and CD4^+^ cells among CD8^+^ cells, and sterility (bacteria, endotoxin and mycoplasma) were analyzed before the release of the products. A detailed description of the CD19/22 CAR T cell structure is provided in **Supplementary Figure 1**.

### Measurement (qPCR)

Based on previous research data, a correlation was observed between the transgene level measured using quantitative polymerase chain reaction (qPCR) and the cell surface expression of CAR in PB samples measured using flow cytometry (**Supplementary Figure 2**). Previous papers identified qPCR as a more sensitive assay than flow cytometry (22, 23). Thus, a qPCR assay was used to detect CD19/22 CAR T cell DNA in PB at various time points (at days 1, 4, 7, 11, 14, 21 and 28 and months 3, 6, 9, 12, 18, 24, 30, 36 and 48) and reported as the number of transgene copies per microgram of genomic DNA. The lower limit of quantitation was 25 copies/μg of genomic DNA.

### Cellular Kinetics (C_max_, T_max_, and AUC)

Cellular kinetics for exposure parameters included the maximal expansion of transgene T cells levels in PB (C_max_) and the time to maximal expansion (T_max_). The area under the curve (AUC) represents the total presence of the cells in both the overall expansion (up to 28 days, AUC_0–28 d_) and persistence (up to 84 days, AUC_0–84 d_) phases after infusion, and persistence was measured by determining the duration of CAR T cells transgene detection in PB [T_last_].

### Effects of Product and Patient Characteristics on Cellular Kinetics

Associations were identified between cellular kinetic parameters and product characteristics, including the T cell percentage, cell viability, transduction efficiency, ex vivo proliferation, and CD4:CD8 ratio. The effects of patient characteristics (age, sex, body weight, prior disease status, and disease stage) and prior clinical treatment factors on cellular kinetics were also analyzed. The effect of the baseline tumor burden (defined by the sum of products of greatest diameters [SPD] or the presence of bulky disease) on cellular kinetics was investigated.

### Relationship Between Cellular Kinetics and Clinical Outcomes

The relationship between cellular kinetic parameters and clinical efficacy (best response status and DOR), as well as between cellular kinetic parameters and adverse events (like CRS and ICANS), were explored. The correlations between cytokine levels and kinetic parameters were also evaluated.

### Statistical Analysis

The cellular kinetics of CD19/22 CAR T cells were calculated using GraphPad Prism 8 (GraphPad software Inc, USA). Associations between cellular kinetics, select product characteristics, and patients’ baseline characteristics were explored using linear models and scatter plots for continuous variables and summary statistics and box plots for categorical variables. Associations between cellular kinetics and the baseline tumour burden were assessed using a linear regression analysis. The effects of cellular kinetics on the response were evaluated using the Mann–Whitney test and a scatter diagram. Effects on DOR were assessed using the Kaplan–Meier method by estimating median values for cellular kinetic parameters. Effects on CRS and neurological events were explored using box plots and a logistic regression analysis. Associations among cellular kinetics, dose and cytokine levels were explored using a linear regression analysis and scatter plots. Analyses of kinetic parameters were exploratory in nature, and the results should be interpreted with caution.

## Results

### Patient Characteristics

From August 2017 to September 2020, a total of 34 patients with R/R B-NHL were enrolled and analyzed. Bispecific CD19/22 CAR T cells were successfully manufactured and administered to 32 patients; in one patient leukapheresis failed, and another could not tolerate the pretreatment because of disease progression. All patients received CAR T cell therapy for the first time and have completed at least 3 months follow-up. All patients who received bispecific CD19/22 CAR T cells were included in the AEs analysis, and 29 patients were included in the efficacy, and the rest of the patients died before reaching the primary efficacy endpoint. A detailed summary of the clinical features of these patients is listed in **Table 1**.

**Table 1:** Patient Demographic and Baseline Disease Characteristics.

Characteristics	No. of Patients, % (32)
Age (years), no. (%)	
<60	24 (75.0)
≥60	8 (25.0)
Sex, no. (%)	
Male	19 (59.4)
Female	13 (40.6)
ECOG perform status score, no. (%)	
0–1	28 (87.5)
2	4 (12.5)
Disease stage at study entry	
III	9 (28.1)
IV	23 (71.9)
Disease type	
DLBCL	27 (84.4)
TFL	2 (6.3)
PMBL	1 (3.0)
HGBL	2 (6.3)
Extranodal organ involvement, no. (%)	
Yes	23 (71.9)
No	9 (28.1)
LDH higher than ULN	15 (46.9)
IPI risk group	
Low (0 or 1 factor)	6 (18.7)
Low/intermediate (2 factors)	7 (21.9)
Intermediate/high (3 factors)	15 (46.9)
High (4 or 5 factors)	4 (12.5)
No. of previous lines of antineoplastic therapy, no. (%)	
<3	19 (59.4)
≥3	13 (40.6)
History of primary refractory	5 (15.6)
Relapsed after HSCT	4 (12.5)
Tumor burden	
SPD ≥100 cm^2^	5 (15.6)
SPD <100 cm^2^	27 (84.4)
Bulky/non-bulky disease	
Lesion diameter ≥10 cm	8 (25.0)
Lesion diameter <10 cm	24 (75.0)

ECOG, Eastern Cooperative Oncology Group; DLBCL, diffuse large B cell; TFL, transformed follicular lymphoma; PMBL, primary mediastinal large B-cell lymphoma; HGBL, high grade B-cell lymphoma; LDH, lactate dehydrogenase; ULN, upper limit of normal; IPI, International Prognostic Index; HSCT, Hematopoietic stem cell transplant; SPD, sum of the product of greatest diameter.

### Clinical Outcomes of R/R B-NHL Patients

**Figure 1:**
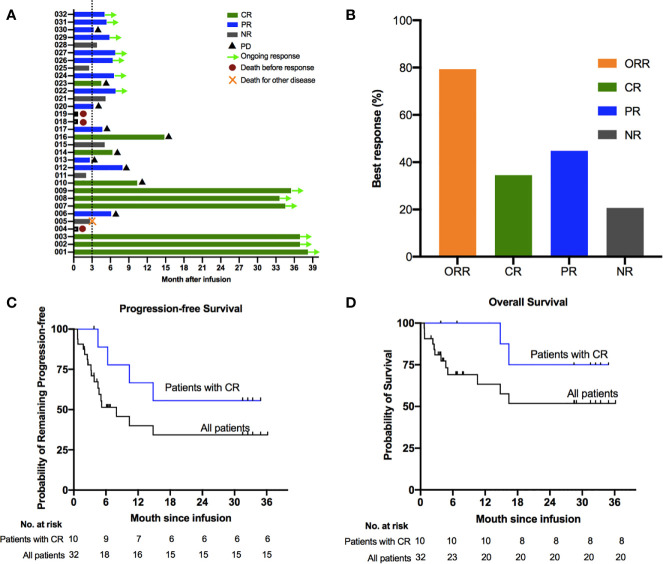
Clinical outcomes of treatment with bispecific CD19/22 CAR T cells. Survival using the Kaplan–Meier method among patients treated with bispecific CD19/22 CAR T cells. **(A, B)** The best overall response rate was 79.3%, and 34.5% of patients achieved a complete response (CR). The median progression-free survival (PFS) was 6.8 months; **(C)** PFS rates were 51.4% at 6 months and 40.0% at 12 months. **(D)** The median overall survival was not reached, with overall survival rates were 69.1% at 6 months and 63.3% at 12 months. **(C, D)** Patients who achieved CR after the CAR T cells infusion experienced prolonged survival compared with those without CR, with the estimated PFS and OS rate at 12 months were 66.7% and 100%, respectively.

Among of 29 patients, the best overall response rate was 79.3%, and 34.5% of the patients achieved a complete response (CR) (**Figures 1A, B**). The data cutoff date for the efficacy evaluation was September 2020, and the corresponding median follow-up time was 8.7 months. The median progression-free survival(PFS) was 6.8 months; the PFS rates were 51.4% at 6 months and 40.0% at 12 months (**Figure 1C**). The median overall survival (OS) was not reached, with rates of 69.1% at 6 months and 63.3% at 12 months (**Figure 1D**). Among patients who reached CR at 3 months, the estimated PFS and OS rates at 12 months were 66.7% and SPD: sum of the product of greatest diameter 100%., respectively (**Figures 1C, D** **)**. Based on the above results, the survival period of patients with CR were longer than that of unreached patients.

At the cut-off data, more than half of the patients remained in remission, while 10/29 patients had disease progression (PD), including seven with DLBCL, two with transformed follicular lymphoma(TFL) and one with high grade B cell lymphoma (HGBL). Among these patients, nine had stage IV disease, seven had international prognostic index(IPI) ≥3 scores, six were positive for Ki-67 in more than 70% of lesion at baseline, and three had PD after ASCT. It was found that 5.3 months was the median time to relapse after CAR T therapy among all patients. The median time to relapse of 4 patients with CR and 6 patients achieved PR was 8.3 months and 3.3 months, respectively.

### Adverse Events

AEs of special interest are summarized in **Table 2**. The most common grade 3 or higher treatment-related AEs (referred to as severe adverse events, sAE) observed within 1 month after infusion included neutropenia (81.3%), anaemia (56.3%) and thrombocytopenia (53.1%). CRS occurred in 29/32 patients (90.6%), including 20/32 (62.5%) patients assessed as grade 1 or 2 and 9/32 (28.1%) as grade 3 or higher. The most common AEs related to severe CRS (grade 3 or higher, sCRS) were hypotension (25.0%), pyrexia (15.6%), and hypoxia (12.5%). The median time from the first infusion of CAR T cells to CRS was 3 days (range 1–11), and the median time to resolution was 5 days. Nine of 29 patients received tocilizumab and 5/29 received glucocorticoids for management of CRS. Most CRS cases ameliorated gradually within 2 weeks after supportive care and tocilizumab or glucocorticoids. One patient died from irreversible, severe CRS-associated acute kidney injury.

**Table 2:** Adverse among All 32 Treated Patients.

Adverse events	Any	Grade 1	Grade 2	Grade 3	Grade 4	Grade 5
Any	30 (93.8)	0	1 (3.1)	7 (21.9)	21 (65.6)	1 (3.1)
CRS	29 (90.6)	14 (43.8)	6 (18.7)	5 (15.6)	3 (9.4)	1 (3.1)
ICANS	5 (15.6)	1 (3.1)	0	4 (12.5)	0	0
Haematological toxicity						
Neutropenia	26 (81.3)	0	0	4 (12.5)	22 (68.8)	0
Thrombocytopenia	26 (81.3)	1 (3.1)	8 (25.0)	8 (25.0)	9 (28.1)	0
Anaemia	30 (93.8)	2 (6.3)	10 (31.3)	14 (43.8)	4 (12.5)	0
General disorders and administration site conditions						
Pyrexia	29 (90.6)	10 (31.3)	14 (43.8)	4 (12.5)	1 (3.1)	0
Fatigue	14 (43.8)	10 (31.3)	4 (12.5)	0	0	0
Chills	13 (40.6)	11 (34.4)	2 (6.3)	0	0	0
Skin rash	2 (6.3)	1 (3.1)	1 (3.1)	0	0	0
Pain	2 (6.3)	2 (6.3)	0	0	0	0
Laboratory tests						
ALT increased	2 (6.3)	0	1 (3.1)	1 (3.1)	0	0
AST increased	3 (9.4)	0	2 (6.3)	1 (3.1)	0	0
T-BIL increased	3 (9.4)	2 (6.3)	1 (3.1)	0	0	0
Creatinine increased	7 (21.9)	2 (6.3)	2 (6.3)	1 (3.1)	1 (3.1)	1 (3.1)
APTT prolonged	12 (33.3)	6 (18.7)	4 (12.5)	2 (6.3)	0	0
Disorders of the Cardiac, respiratory system, renal system, and Gastrointestinal system						
Hypotension	13 (40.6)	4 (12.5)	1 (3.1)	7 (21.9)	1 (3.1)	0
Hypoxia	7 (21.9)	3 (9.4)	0)	4 (12.5)	0	0
Heart failure	2 (6.3)	0	0	0	2 (6.3)	0
Dyspnoea	2 (6.3)	0	1 (3.1)	1 (3.1)	0	0
Acute kidney injury	2 (6.3)	0	0	0	1 (3.1)	1 (3.1)
Nausea	7 (21.9)	3 (9.4)	4 (12.5)	0	0	0
Vomiting	8 (25.0)	5 (15.6)	3 (9.4)	0	0	0
Abdominal distention	4 (12.5)	2 (6.3)	1 (3.1)	1 (3.1)	0	0
Diarrhoea	4 (12.5)	4 (12.5)	0	0	0	0
Infections						
Lung infection	5 (15.6)	0	0	5 (15.6)	0	0
Septicaemia	2 (6.3)	0	0	2(6.3)	0	0
Neurologic events						
Delirium	2 (6.3)	0	1 (3.1)	1 (3.1)	0	0
Epilepsy	2 (6.3)	0	1 (3.1)	1 (3.1)	0	0
Somnolence	2 (6.3)	0	1 (3.1)	1 (3.1)	0	0
Cognitive disturbance	3 (9.4)	0	1 (3.1)	2 (6.3)	0	0
Speech disorder	1 (3.1)	0		1 (3.1)	0	0

Severity of adverse events was graded according to the National Cancer Institute Common Terminology Criteria for Adverse Events, version 5.0. Cytokine release syndrome and neurologic events were graded according to the American Society for Blood and Marrow Transplantation (ASBMT) consensus. Regarding the grade 5 events, one patient died from acute kidney injury related to CAR T therapy. CRS, cytokine release syndrome; ICANS, immune effector cell-associated neurotoxicity syndrome; ALT, alanine aminotransferase; AST, aspartate aminotransferase; T-BIL, total bilirubin; APTT, activated partial thromboplastin time.

Neurologic events occurred in five patients (15.6%), and four patients were assessed as having grade 3 or higher ICANS (sICANS). The most common sICANS was cognitive disturbance (6.3%). The median time from the first infusion of CAR T cells to ICANS was 11.5 days (range 8–20). In four of five patients ICANS resolved within 1 week with glucocorticoids and supportive treatment, and the remaining patient died due to severe CRS-associated acute kidney injury before ICANS resolved.

### Cellular Kinetics

Cellular kinetic data were analysed for 32 patients. Summary results of cellular kinetics obtained using qPCR are shown in **Table 3**. The cellular kinetic profile of CAR T cells in PB was described as three phases: “distribution”, “expansion”, and “persistence”. In the distribution phase, the concentration of CAR T cells decreased due to the distribution of CAR T cells from PB into various tissues and organs after their infusion, which has been reported by other researchers (2, 17, 27, 28). This stage is usually observed 0–5 days after the CAR T cells infusion. In the expansion phase, a rapid increase in the number of CAR T cells in the peripheral circulation is noted, followed by the C_max_ and then a rapid decline. This stage is usually observed 6–28 days after the CAR T cells infusion, and CAR T cells levels in PB typically peak at days 10 to 14 postinfusion. After day 28, infused CAR T cells undergo the persistence phase, and the number of CAR T cells remains constant and decreases slowly.

**Table 3:** Summary of cellular kinetic parameters stratified by response.

Parameters	CR/PR	PD/SD/Unknown	All Patients	
	N = 23	N = 9	N = 32	
AUC_0–28d_ (copies/μg*days)				
N	23	6	29	
Geometric mean	1,635,220.0	564,240.7	1,212,189.4	
CV%	110.3	158.4	124.8	
Fold difference (responders vs non-responders)	289%			
AUC_0–84 d_ (copies/μg*days)				
N	18	4		22
Geometric mean	1,971,703.9	752,917.4		1,655,101.1
CV%	113.8	61.9		122.6
Fold difference (responders vs non-responders)	262%			
C_max_ (copies/μg)				
N	23	9		32
Geometric mean	331,312.1	197,118.8		286,294.4
CV%	107.1	272.8		346.8
Fold difference (responders vs non-responders)	168%			
T_max_ (days)				
n	23	9		32
Median	12	12		12
Min, max	2, 25	1, 28		1, 28
T_last_ (days)				
n	23	9		32
Median	133	22		92.5
Min, max	15, 763	13, 174		13, 763

CR, complete response; PR, partial response; PD, progressive disease; SD, stable disease; AUC, area under the curve; CV, coefficient of variation; C_max_, maximal expansion of transgenic T cell levels in peripheral blood after the infusion; T_max_, time to maximal expansion; T_last_, T cells are present in peripheral blood.

### Effect of the Characteristics of CD19/22 CAR T Cells on Cellular Kinetics

We analyzed the correlation between cellular kinetics and characteristics of CD19/22 CAR T cells after expansion to evaluate the effect of characteristics of CD19/22 CAR T cells on cellular kinetics. Our data did not reveal apparent relationships between cellular kinetics and product characteristics according to the currently established release standards (**Supplementary Figure 3**).

### Effect of Patient Characteristics on Cellular Kinetics

**Figure 2:**
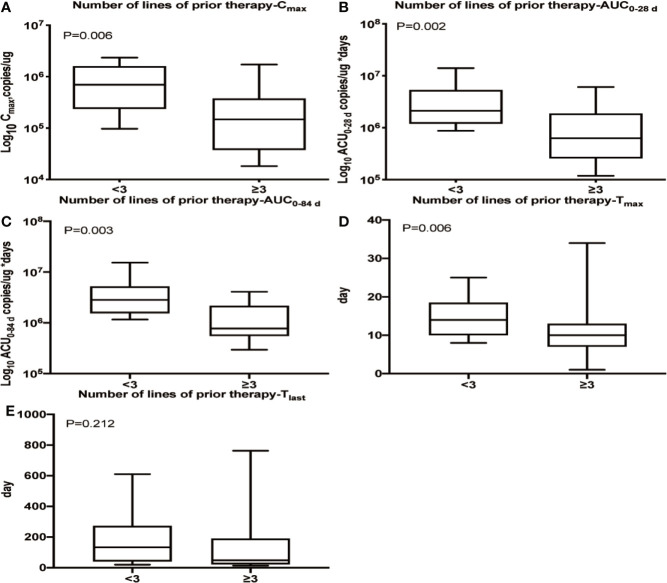
Relationship between the number of prior treatment lines and cellular kinetics. **(A)** Patients who had received three or more lines of prior treatment had a lower C_max_ (P = 0.006). **(B)** Patients who had received three or more lines of prior treatment had a lower AUC_0–28 d_ (P = 0.002). **(C)** Patients who had received three or more lines of prior treatment had a lower AUC_0–84 d_ (P = 0.003). **(D)** Patients who had received three or more lines of prior treatment had a lower T_max_ (P = 0.006). **(E)** No difference was observed in T_last_ among patients with different numbers of lines of prior treatment (P = 0.212).

We observed that patients who had received three or more lines of prior treatment had lower C_max_ (P = 0.006), T_max_ (P = 0.006), AUC_0–28 d_ (P = 0.002) and AUC_0–84 d_ (P = 0.003) values than patients with less than three lines of prior treatment, but no difference was observed in T_last_ (**Figure 2**). Additionally, a lower C_max_ was observed in patients with extranodal organ involvement than in patients without extranodal organ involvement (P = 0.010, **Supplementary Figure 4**), while T_max_, AUC_0–28 d_, AUC_0–84 d_ and T_last_ were nearly identical between the two subpopulations (data not shown). No apparent relationships were identified between cellular kinetics and other selected baseline characteristics of patients (**Supplementary Figure 4**).

### Efficacy and Cellular Kinetics

**Figure 3:**
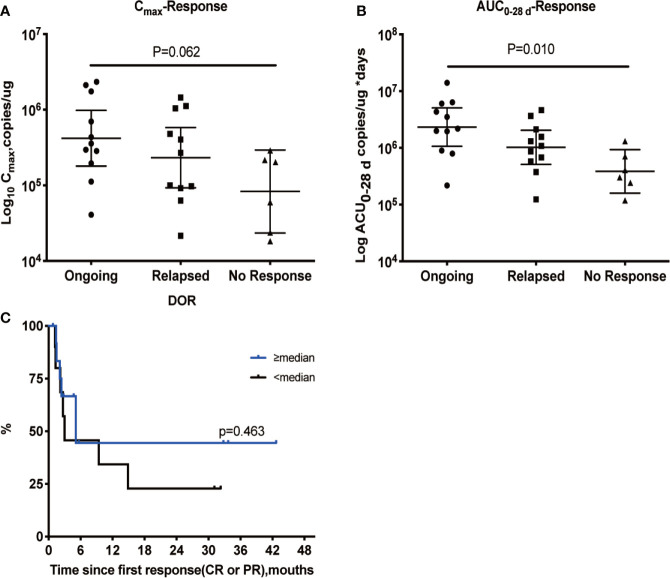
Relationship between cellular kinetics and response. Relationship between C_max_ **(A)** and AUC_0–28 d_ **(B)**. DOR in patients with a response **(C)**. Patients with an ongoing response at the last follow-up day had higher C_max_ **(A)** and AUC_0–28 d_ values **(B)**. Patients with a higher-than-median C_max_ had a potentially longer DOR than those with lower-than-median C_max_, but the differences were not statistically significant (P = 0.463) **(C)**.

Based on the cellular kinetic profile described above, important PB kinetic parameters were calculated for all available patients. Median maximal expansions and exposures during the first 28 and 84 days following infusion of patients with CR and partial response (PR) were significantly higher than non-responding (NR) patients, suggesting that responders tended to have higher expansion of CAR T cells in PB than non-responders. The geometric mean C_max_ of CR/PR patients was 331,312.1 copies/µg, which was 168% higher than of NR patients, and the geometric mean C_max_ of NR patients was 197,118.8 copies/µg. Exposure during the first 28 and 84 days (AUC_0–28 d_ and AUC_0–84 d_) values were higher in CR/PR patients than NR patients (289% higher for AUC_0–28 d_ and 262% higher for AUC_0–84 d_) (**Table 3**). In parallel, the median T_last_ detected using qPCR in responders (133 days) was longer than that in non-responders (22 days) (P = 0.004). The median T_max_ was also calculated and compared between the two subpopulations, and no difference was found (P = 0.864). In addition, patients with an ongoing response at the last follow-up day had higher C_max_ and AUC_0–28 d_ values (**Figures 3A, B**), but not AUC_0–84 d_ (data not shown). Patients with a higher-than-median C_max_ had a potentially longer DOR versus those with a lower-than-median C_max_ (**Figure 3C**), but the differences were not statistically significant (P = 0.463).

### Safety and Cellular Kinetics

**Figure 4:**
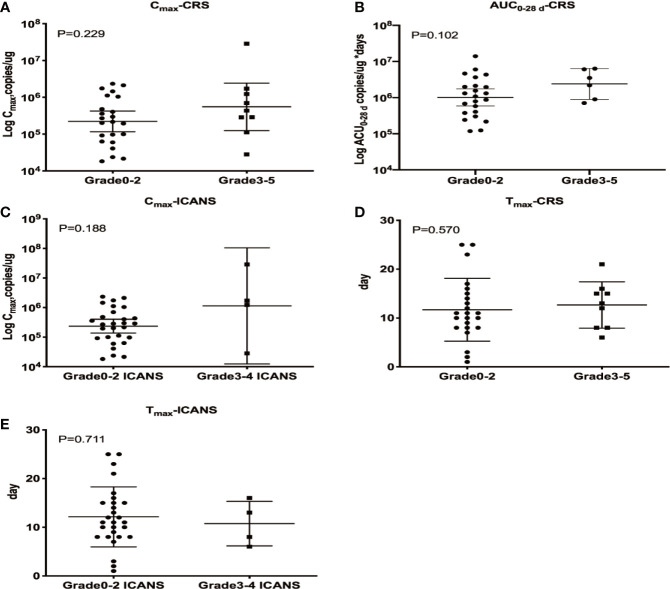
Relationship between cellular kinetics and AEs. Relationships between C_max_ and CRS **(A)**, AUC_0–28 d_ and CRS **(B)**, C_max_ and ICANS **(C)**, T_max_ and CRS **(D)** and T_max_ and ICANS **(E)**. **(A, B)** Patients with sCRS exhibited higher CD19/22 CAR T C_max_ and AUC_0–28 d_ values than those with grade 0–2 CRS, but these differences were not statistically significant (P = 0.229 and P = 0.102, respectively). **(C)** Patients with sICANS exhibited a higher C_max_ than those with low/no ICANS, but this difference was not statistically significant (P = 0.188). **(D, E)** Neither CRS nor ICANS was correlated with T_max_ (P = 0.570 and P = 0.711, respectively).

CRS and ICANS were the most common AEs reported in patients undergoing CAR T cell therapy, and the correlations between these AEs and CAR T cells PB kinetics were investigated. Patients with sCRS exhibited higher CD19/22 CAR T C_max_ and AUC_0–28 d_ values than those with grade 0–2 CRS, but these differences were not statistically significant (P = 0.229 and P = 0.102, respectively), indicating that higher levels of CAR T cells expansion in PB may be correlated with more severe CRS. However, C_max_ and AUC_0–28 d_ were not associated with an increased estimated probability of any-grade or grade 3/4 CRS based on the results of logistic regression models. All four patients with sICANS developed sCRS, and patients with sICANS exhibited a higher C_max_ than those with low/no ICANS, but this difference was not statistically significant (P = 0.188). The determination of AUC_0–28 d_ using qPCR requires at one sample with detectable levels beyond 28 days after CAR T cell therapy, which were only available for one patient with ICANS. Therefore, this parameter was not assessed in patients with ICANS. Neither CRS nor ICANS was correlated with T_max_ (**Figure 4**).

### Effect of the Dose on Cellular Kinetics, Efficacy and Safety

**Figure 5:**
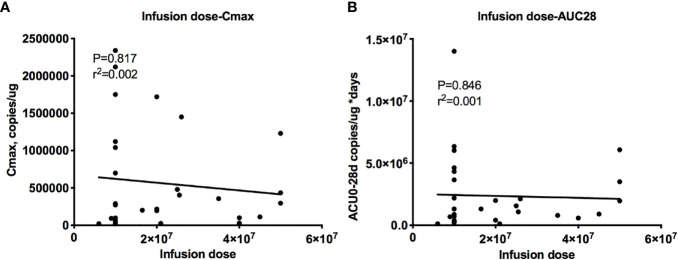
Relationship between the dose and cellular kinetics. Relationships between the infusion dose and C_max_ **(A)** and AUC_0–28 d_ **(B)**. The dose-exposure analysis showed a flat relationship between the dose and cellular kinetic parameters, with r^2^ = 0.004 for C_max_ **(A)** and r^2^ = 0.002 for AUC_0–28 d_ **(B)**.

The dose-exposure analysis showed a flat relationship between the dose and cellular kinetic parameters, with r^2^ = 0.004 for C_max_ and r^2^ = 0.002 for AUC_0–28 d_ (**Figure 5**), and the dose did not affect the efficacy or safety (data not shown). Therefore, CAR T cells have the ability to undergo a rapid exponential amplification beyond the initial infused dose, and no relationships between the dose and peak expansion, exposure, efficacy and safety were observed across a wide dose range (3.690 ∗ 10^8^ to 3.285 ∗ 10^9^ cells) (data not shown).

### Effect of Tumor Burden on CD19/22 CAR T Cell Expansion, Efficacy, CRS and ICANS

**Figure 6:**
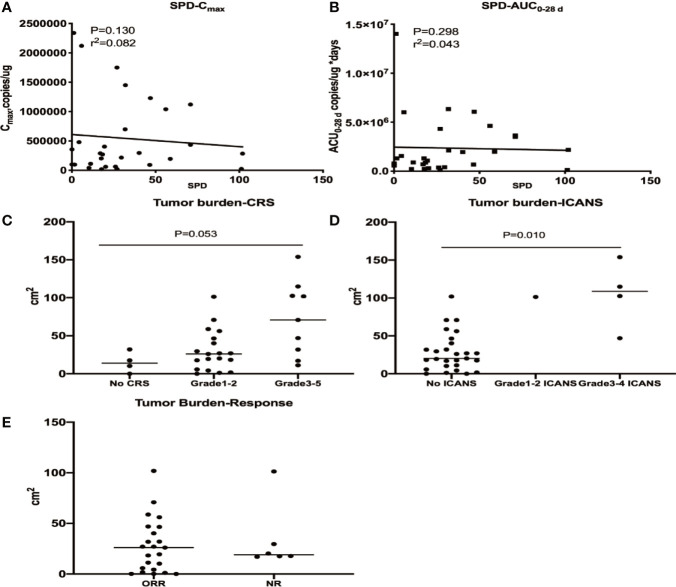
Relationships between the tumour burden and cellular kinetics, CRS, ICANS and efficacy. The baseline tumor burden did not affect C_max_ **(A)** and AUC _0–28 d_ **(B)**. A higher tumor burden was observed in patients with grade ≥3 CRS **(C)** and ICANS **(D)**, while no apparent correlation was identified between the tumor burden and response **(E)**.

The baseline tumor burden did not affect cellular kinetic parameters. A higher tumour burden was observed in patients with grade ≥3 CRS and ICANS, while no apparent correlation was identified between the tumor burden and response (**Figure 6**). A multivariate analysis was performed to evaluate the effects of the C_max_, dose, and tumour burden on the probability of high-grade CRS and ICANS and showed that tumour burden was correlated with grade ≥3 CRS (OR 1.032, P = 0.027) but not grade ≥3 ICANS (OR 1.098, P = 0.227) (**Supplementary Table 1**).

### Cellular Kinetics and Serum Cytokine Levels

Serum cytokine levels were measured in all patients. Levels of cytokines such as IL-6 increased after the infusion and remained elevated in patients experiencing CRS. No correlation was identified between C_max_ and cytokine levels during the first 28 days (**Supplementary Figure 5**). Patients with ≥3 grade CRS generally had higher cytokine levels (**Supplementary Figure 6**).

## Discussion

Numerous studies have shown that CD19 CAR T cell therapy is an effective treatment for R/R B-NHL. However, this therapy is also associated with a high rate of relapsed among patients with R/R B-NHL and R/R ALL (2–7). Simultaneous targeting of more than one B cell antigen has been proposed as a therapeutic strategy to reduce the risk of relapse mediated by antigen-negative clonal escape (8, 29, 30). Recently, studies have found that bispecific CD19/20 CAR T cells are feasible and therapeutically safe, showing low toxicity and high efficacy in R/R B-NHL patients (12, 31). CD22 is expressed in both normal B-cell and associated malignancies, and expressed at the late pre-B-cell stage but not found in hematopoietic stem cells (HSCs) (32), constituting a promising targets for B-NHL. Thus, we designed the bispecific CD19/22 CAR T cells, involving a loop CAR molecule and consisting of an anti-CD22 scFv derived from mouse m971 mAb and anti-CD19 scFv derived from the mouse FMC63 mAb, joined in the loop, a human CD8α hinge and transmembrane domain, and human 4-1BB and CD3ζ signaling domains. We speculate that bispecific CD19/22 CAR T cells are able to be stimulated by many more antigens in PB and have better antitumor activity than CD19 CAR T cells, because of the bispecific CD19/22 CAR T cells could not only recognize and kill CD19^+^ tumor cells but also recognize CD22^+^ and CD19^+^CD22^+^ tumor cells. Here, we report the clinical outcomes and first comprehensive analysis of the pharmacokinetics of a clinical trial for bispecific CD19/22 CAR T cells for patients with R/R B-NHL.

In this study, we present the safety and efficacy of treatment with bispecific CD19/22 CAR T cells for R/R B-NHL, with a similar rate of 3–4 CRS and ICANS and response rate as CD19 CAR T cells. Nasheed et al. treated seven patients with relapsed/refractory B cell malignant with bispecific CD19/22 CAR T cells; two patients (28.6%) achieved CR and three others (42.9%) achieved PR (33). Recently, one study showed the development of CD19-relapsed was not eliminated in R/R ALL patients treated with CD22 CAR T cell therapy who relapsed after CD19 CAR T cell therapy (34). However, other groups have focused on dual targeting of CD19 and CD20 on malignant B cells, reporting improved safety and response rates (12, 31). Thus, further investigation is needed to determine the optimal CAR T targets for B cell NHL.

Before CAR T cell treatment, patients usually suffer from a refractory or relapsed disease and have received heavy treatment with multiple lines of chemotherapies. Due to those prior treatments, patients often experience bone marrow (BM) suppression, reduced immune cell function and BM microenvironmental damage. Although the immune system can recover, this process takes a long time and the immune system of patients with R/R B-NHL is not easily restored to its normal state after multiple treatments. Moreover, the more treatment lines the patient has received in the previous period, the greater damage to the patient’s immune system. Solomayer and colleagues reported that chemotherapy exerts a particularly suppressive effect on naïve CD4 T cells and, to a lesser extent, on memory CD4 T cells in patients with breast cancer (35). In fact, many reports have documented that patients treated with CAR T cells enriched for naïve and stem central memory cells expanded well in PB (36–38). As expected, our study reported lower C_max_, AUC_0–28 d_ and AUC_0–84 d_ values in patients who had received three or more lines of prior treatment than in those treated with less than three lines of prior treatment. We speculate that one reason for the low expansion of CAR T cells in patients who previously received extensive treatment in our study is that the administration of multiple lines prior to CAR T cell treatment damaged T cell function and reduced naïve and memory T cell numbers, leading to the poor expansion and persistence of CAR T cells, which requires a further analysis of the subtypes of CAR T cells to verify this hypothesis. However, a trial with Tisagenlecleucel in patients with DLBCL showed contrasting results, as the number of lines of prior therapies was not associated with peak expansion and the AUC (24). Due to the structure and targets of CAR T cells, the product characteristics and patient population in the two studies were not consistent, which may have resulted in different kinetic behaviors.

Disease characteristics often affect the pharmacokinetics and clinical response of patients with B-NHL treated with traditional cytotoxic chemotherapies, most likely because some of these attributes are associated with chemotherapy metabolism and resistance (39–41). In patients with extranodal organ involvement, the C_max_ and T_max_ values of CD19/22 CAR T cells in PB were lower than those of patients without extranodal involvement in the present study, but extranodal involvement did not alter AUC and T_last_. We are not the first group to report this finding; others have shown that CAR T cells traffic from PB into various tissues and organs after the infusion, which has been confirmed by assessing the transgene levels expressed in CAR T cells in other tissues, including BM and cerebrospinal fluid (2, 17, 27, 28). The findings from our study and other researchers indicate that the extranodal lesions of NHL require CAR T cells to transfer from PB to the extranodal lesions to kill tumor cells, which alter the time to peak and the speed of maximum peripheral blood concentration. While, the distribution of CAR T cells to extranodal lesions does not affect CAR T cells expansion and persistence. CAR T cells trafficking from PB to tissues or organs in patients with B-NHL may result in a longer half-life compared with patients with B-ALL, where the target is predominantly in the blood (24).

Correlations between pharmacokinetic parameters and clinical efficacy were investigated. We noticed greater expansion and persistence in responders than in non-responders, consistent with a single-center study of CD19 CAR T cells in patients with large B cell lymphoma (6). A similar correlation was also observed between cellular kinetics and clinical efficacy in patients with CLL and B-ALL treated with CD19 CAR T cells (17, 23, 42). In contrast, no differences in expansion and persistence were observed between responders and non-responders in the JULIET study of CD19 CAR T cells for R/R B-NHL (24). However, patients with a C_max_ higher than the median had a longer DOR than patients with a lower-than-median C_max_ in that study (24). Similarly, our data also suggested that patients with a C_max_ higher than the median C_max_ had a longer DOR than those with lower-than-median C_max_ values. In parallel, most responders had a longer T_last_ than non-responders. However, the time of persistence may be influenced by the data cut-off and length of follow-up, with limited follow-up generally observed in non-responders; therefore, a comparison between responders and non-responders should be performed with caution. Notably, patients with an ongoing response at the last follow-up time had higher CAR T cells peak concentrations and AUC values in the first 28 days after the CD19/22 CAR T cells infusion than other patients, suggesting that maximal expansion may have a greater contribution to a longer DOR in responders than transgene persistence. This finding is consistent with a previous public study of patients with R/R B-NHL showing that an ongoing response is associated with high CAR T cells peak concentrations and AUC values in the first 28 days after the CAR T cell infusion (9).

Analyses of CAR T kinetics and safety issues were performed. Our study found that grade 3/4 CRS was associated with higher C_max_ (P = 0.229) and AUC_0–28 d_ (P = 0.102) values than no/low-grade CRS, consistent with other reports (17, 23, 27). Awasthi et al. studied the effectiveness of Tisagenlecleucel in patients with relapsed/refractory NHL and found a trend toward higher CAR T cells expansion with increasing CRS severity (24). Data on CAR T cells therapy from the Fred Hutchinson Cancer Research Center revealed that higher peak CAR T cells numbers were associated with high-grade CRS in patients with relapsed/refractory B-ALL, CLL, or NHL (43, 44). Thus, CRS is correlated with the acute expansion of CAR T cells in PB and is correlated with CRS severity. Our data showed a strong correlation between CRS and ICANS, but data were only available for one patient with ICANS who had sufficient samples to analyze AUC_0–28 d_; therefore, the correlation was not assessed between cellular kinetics and ICANS. Importantly, an association between the baseline tumor burden and CRS severity was observed, consistent with previous studies of Tisagenlecleucel in patients with ALL and B-NHL; patients with a high tumour burden were at an increased risk of developing higher-grade CRS and had greater PB Tisagenlecleucel expansion than those with a low tumour burden (17, 24). In contrast, relationships between the baseline tumour burden and CRS were also not observed in patients with B-NHL treated with Axicabtagene Ciloleucel (6, 45). The inconsistency of the observations noted in different studies of CAR T cell therapy in patients with B-NHL may be attributed to differences in the target population, costimulatory domains, reported units, or structure of CAR T cells.

We analyzed the correlation between cellular kinetics and some characteristics of CD19/22 CAR T cells after expansion to evaluate the effect of the characteristics of CD19/22 CAR T cells on cellular kinetics. Our data did not reveal apparent relationships between cellular kinetics and selected CAR T product characteristics, consistent with the results obtained for Tisagenlecleucel single target CD19 CAR T cells in patients with R/R DLBCL (24). This finding provided clinical justification for specifying the range for these characteristics and indicated that CAR T cells have the potential to expand in PB, regardless of the CAR T product characteristics. However, because data on T cell subtypes in the CD19/22 CAR T cells population are lacking, further research is needed to determine how the subtypes of CAR T cell products affect cellular kinetics.

Our study has several limitations. Proliferation and persistence of CAR T cells are the key factors for anti-tumor efficacy and safety after infusion, so it is important to detect circulating CAR T cells in PB. Approaches such as qPCR which detect the integrated CAR transgene sequence, and flow cytometry which detect the CAR antigen expression have been developed. Many studies have demonstrated that the level of CAR T cells detected by qPCR correlated well with the level of CAR-positive cells detected by flow cytometry. In our study, we used qPCR method to detect the cellular kinetic parameters. However, qPCR cannot specifically characterize the ratio of CD4^+^ and CD8^+^ T cell in viable cells. And in our study, we did not fully evaluate the factors that may affect the proliferation and persistence of CAR T cells. For example, the T cell phenotypes of CAR T cells, tumor micro-environment, patients’ own T cell characterization, and patients’ tumor cells characterization. Although previous researches have reported that lymphoma exhibited lower antigen-negative relapsed rates compared with those in leukemia after CAR T treatment, it is worthwhile to detect the relapse phenotype. However, due to the inability to obtain biopsy tissue, we were unable to obtain this data.

In summary, along with our findings, the bispecific CD19/22 CAR T cells have a favorable toxicity profile and efficacy in R/R B-NHL, and the responders showed a higher maximum concentration and prolonged long-term persistence of CAR T cells in PB. In addition, the expansion of CAR T cells in the first 28 days was associated with CRS, which is an important AE. Our research reveals the safety and potential clinical efficacy of bispecific CD19/22 CAR T cells in patients with R/R B-NHL and emphasizes that measuring PB kinetic parameters is useful for predicting the efficacy and safety in clinical applications of CAR T cell therapy.

## Data Availability Statement

The original contributions presented in the study are included in the article/**Supplementary Material**. Further inquiries can be directed to the corresponding author.

## Ethics Statement

The studies involving human participants were reviewed and approved by the Institutional Ethics Committee of the First Affiliated Hospital of Soochow University. The patients/participants provided their written informed consent to participate in this study.

## Author Contributions

YZ, JL, and XL analyzed data and wrote the manuscript. XC, JC, JZ, XZ, ZYa, SJ, HG, GC, HD, and XT were the clinicians who participated in the treatment of the patient. LK, ZYu, ML, NX, and LY designed and manufactured the CAR T cells. DW and CL designed the clinical protocol. DW was the principal investigator of the trial. CL was the secondary investigator of the trial. YZ, JL, XL, DW, and CL contributed equally to this study. All authors contributed to the article and approved the submitted version.

## Funding

The authors acknowledge the following funders: National Natural Science Foundation of China (81730003), National Science and Technology Major Project (2017ZX09304021), Priority Academic Program Development of Jiangsu Higher Education Institutions (PAPD), and Science Planning Project of Suzhou (sys2018049).

## Conflict of Interest

XL, ZYu, LK, ML, NX, and LY are employees of Shanghai Unicar-Therapy Bio-medicine Technology Co., Ltd.

The remaining authors declare that the research was conducted in the absence of any commercial or financial relationships that could be construed as a potential conflict of interest.
