# Supplementary material for: A Prospective Investigation of Bispecific CD19/22 CAR T Cell Therapy in Patients With Relapsed or Refractory B Cell Non-Hodgkin Lymphoma
Source: Front Oncol. 2021 May 25;11:664421. doi: 10.3389/fonc.2021.664421 (PMC8185372; doi:10.3389/fonc.2021.664421)
Supplement: Supplementary file 1 [file DataSheet_1.zip › Additional materials/Supplement Figure Captions.pdf]

SUPPLEMENT FIGURE CAPTIONS

Supplementary Figure 1. A schematic of the CD19/CD22 CAR T. CD19/22 CAR T was a loop CAR molecule, consisting of an anti-CD22 scFv derived from mouse m971 mAb and anti-CD19 scFv derived from the mouse FMC63 mAb, joined in the loop, human CD8α hinge and transmembrane domain, and human 4-1BB and CD3ζ signaling domains.

Supplementary Figure 2. Correlation between qPCR and flow cytometry. A correlation was observed between the transgene level from qPCR and the cell surface expression of CAR from flow cytometry in PB summarized both by individual patient C_{max} (r = 0.612; P =0.033) and AUC_{0-28d} (r =0.758; P =0.008) values.

Supplementary Figure 3. Relationship between product characteristics and cellular kinetics. Relationship between percentage of T cells (A), cell viability (B), transduction efficiency by qPCR (C), cell expansion fold in vitro(D), and CD4:CD8 ratio vs AUC0-28d (E).

Supplementary Figure 4. Relationship between patient's characteristics and cellular kinetics. Relationship between age (A), weight (B), sex (C), refractory disease(D), prior HSCT status(E), extranodal organ involvement(F)and Bulky disease vs C_{max} (G).

Supplementary Figure 5. Relationship between cytokines and cellular kinetics. There was no correlation between C_{max} and cytokine levels during the first 28 days.

Supplementary Figure 6. Relationship between cytokines and Cytokine release syndrome(CRS). Patients with ≥3 grade CRS generally had higher levels of cytokines.
