# Supplementary material for: A Prospective Investigation of Bispecific CD19/22 CAR T Cell Therapy in Patients With Relapsed or Refractory B Cell Non-Hodgkin Lymphoma
Source: Front Oncol. 2021 May 25;11:664421. doi: 10.3389/fonc.2021.664421 (PMC8185372; doi:10.3389/fonc.2021.664421)
Supplement: Supplementary file 1 [file DataSheet_1.zip › Additional materials/Supplement-total file.pdf]

Supplementary Methods

Eligibility criteria

(1) Histological detection confirmed non-Hodgkin lymphoma with measurable criteria

(2) Recieved more than 2 lines of chemotherapy

(3) With contraindications of hematopoietic stem cell transplantation or relapse after hematopoietic stem cell transplantation

(4) Age$\geqslant$18 and Age<78 years old

(5) Expected survival more than 3 months

(6) Karmofsky performance score $\leqslant$ 60, and ECOG $\geqslant$ 2

(7) Enough organ function: EF$\geqslant$50%; normal ECG; CCR$\geqslant$40ml/min; ALT and AST $\leqslant$ 3 × upper limitation of normal, T-BIL $\leqslant$ 2.0mg/dl; PT and APTT < 2 × upper limitation of normal; SpO2 > 92%

(8) CBC results: Hb $\geqslant$ 80g/L, ANC > 1 × 10^9/L, Plt $\geqslant$ 50 × 10^9/L

(9) Results of pregnant test should be negative, and agree to conception control during treatment and 1 year after CAR-T infusion

(10) Provided written informed consent before any screening procedures

Exclusion criteria

(1) Received immunosuppression treatment or steroids in recent 1 week before recruitment

(2) Uncontrolled infection

(3) HIV positive patients

(4) Active HBV or HCV infection

(5) Women in pregnancy and lactation

(6) Refuse to conception control during treatment and 1 year after CAR T infusion

(7) Uncured malignancies other than non-Hodgkin lymphoma

(8) Have participated similar trial for treating relapse/refractory non-Hodgkin lymphoma

(9) Inheritated immune deficiancy

(10) Severe heart disease

Supplementary Table1. Multivariate Analaysis of Impact of Expansion(C_{max}), Dose, and Tumor Burden on Probability of Grad ≥3 CRS and ICANS

	Grade ≥3 CRS		Grade≥3 ICANS	
	Parameter estimate	P value	Parameter estimate	P value
Log(Dose)	2.310	0.330	11.035	0.465
Log(C_{max})	13.335	0.152	54326.262	0.256
Tumor burden	1.032	0.027	1.098	0.227

C_{max}: maximal expansion of transgene T-cell levels in peripheral blood post infusion; CRS: cytokine release syndrome; ICANS: immune effector cell-associated neurotoxicity syndrome.

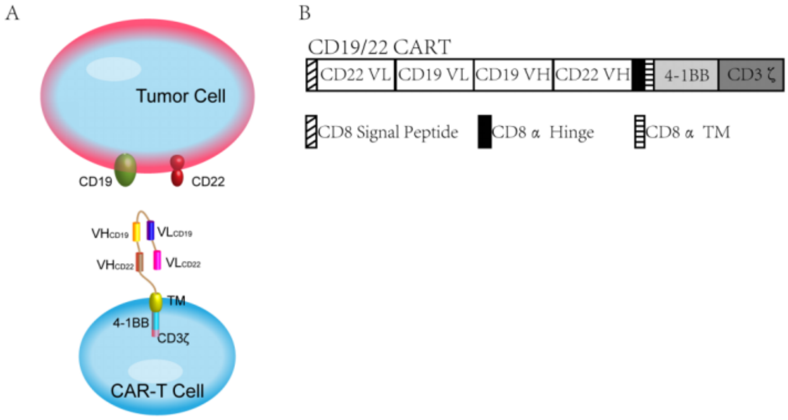

Supplementary Figure 1. A schematic of the CD19/CD22 CAR T. CD19/22 CAR T was a loop CAR molecule, consisting of an anti-CD22 scFv derived from mouse m971 mAb and anti-CD19 scFv derived from the mouse FMC63 mAb, joined in the loop, human CD8α hinge and transmembrane domain, and human 4-1BB and CD3ζ signaling domains.

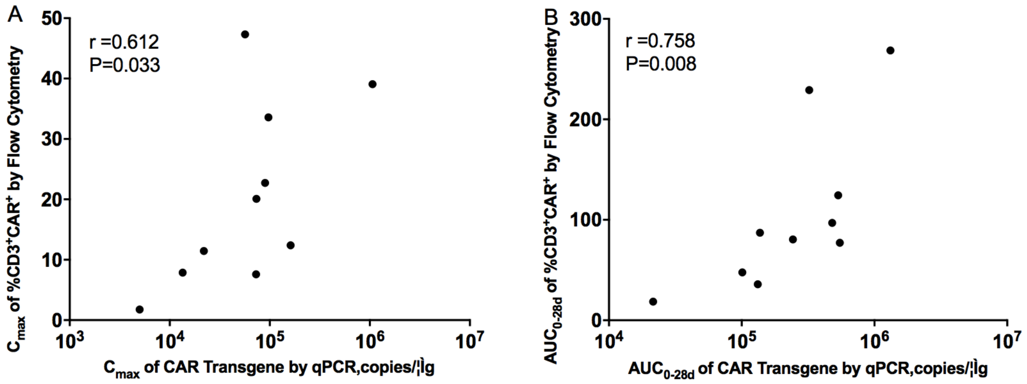

Supplementary Figure 2. Correlation between qPCR and flow cytometry. A correlation was observed between the transgene level from qPCR and the cell surface expression of CAR from flow cytometry in PB summarized both by individual patient C_{max} ($r = 0.612$; $P = 0.033$) and $AUC_{0\text{-}28d}$ ($r = 0.758$; $P = 0.008$) values.

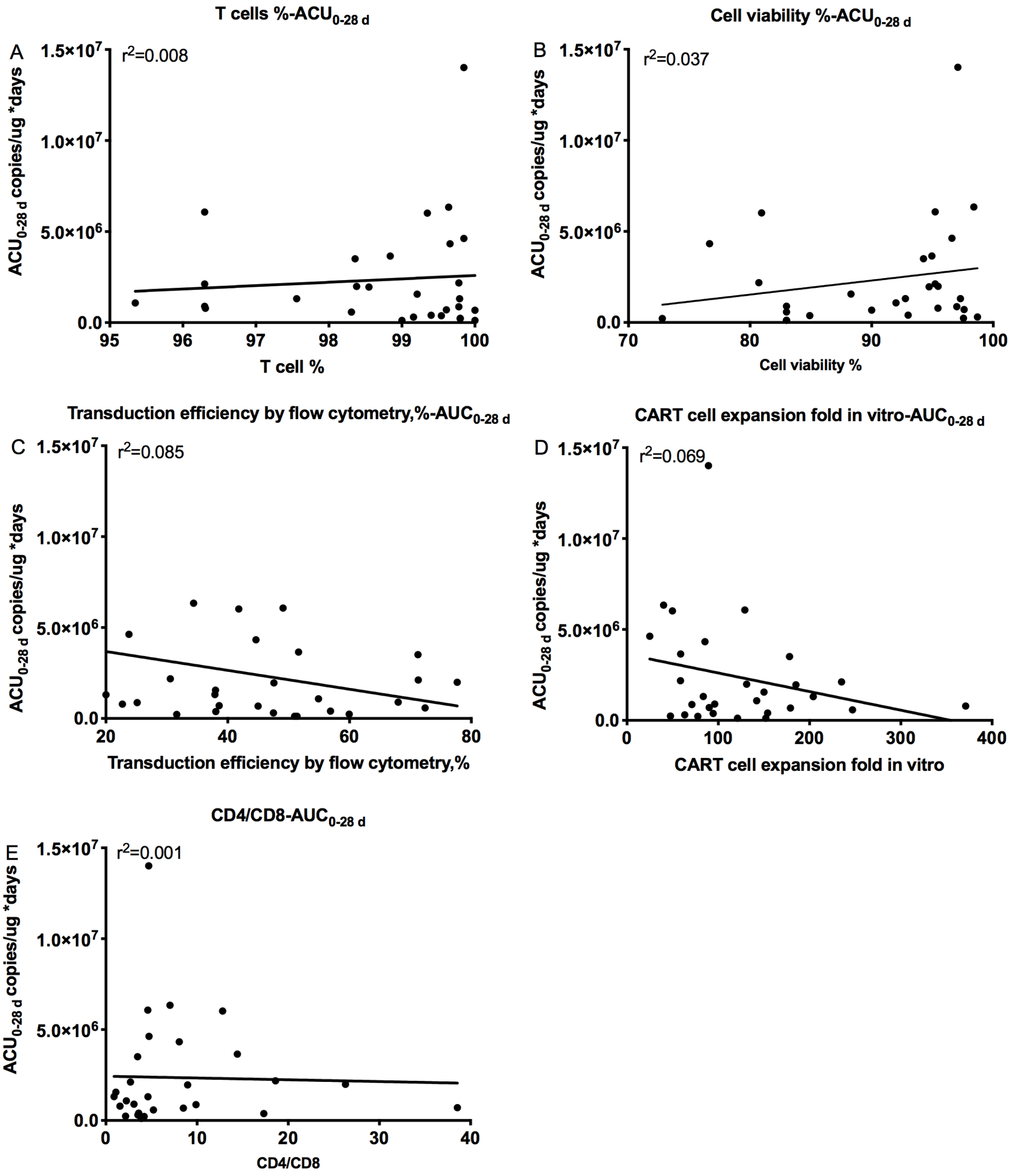

Supplementary Figure 3. Relationship between product characteristics and cellular kinetics. Relationship between percentage of T cells (A), cell viability (B), transduction efficiency by qPCR (C), cell expansion fold in vitro(D), and CD4:CD8 ratio vs AUC0-28d (E).

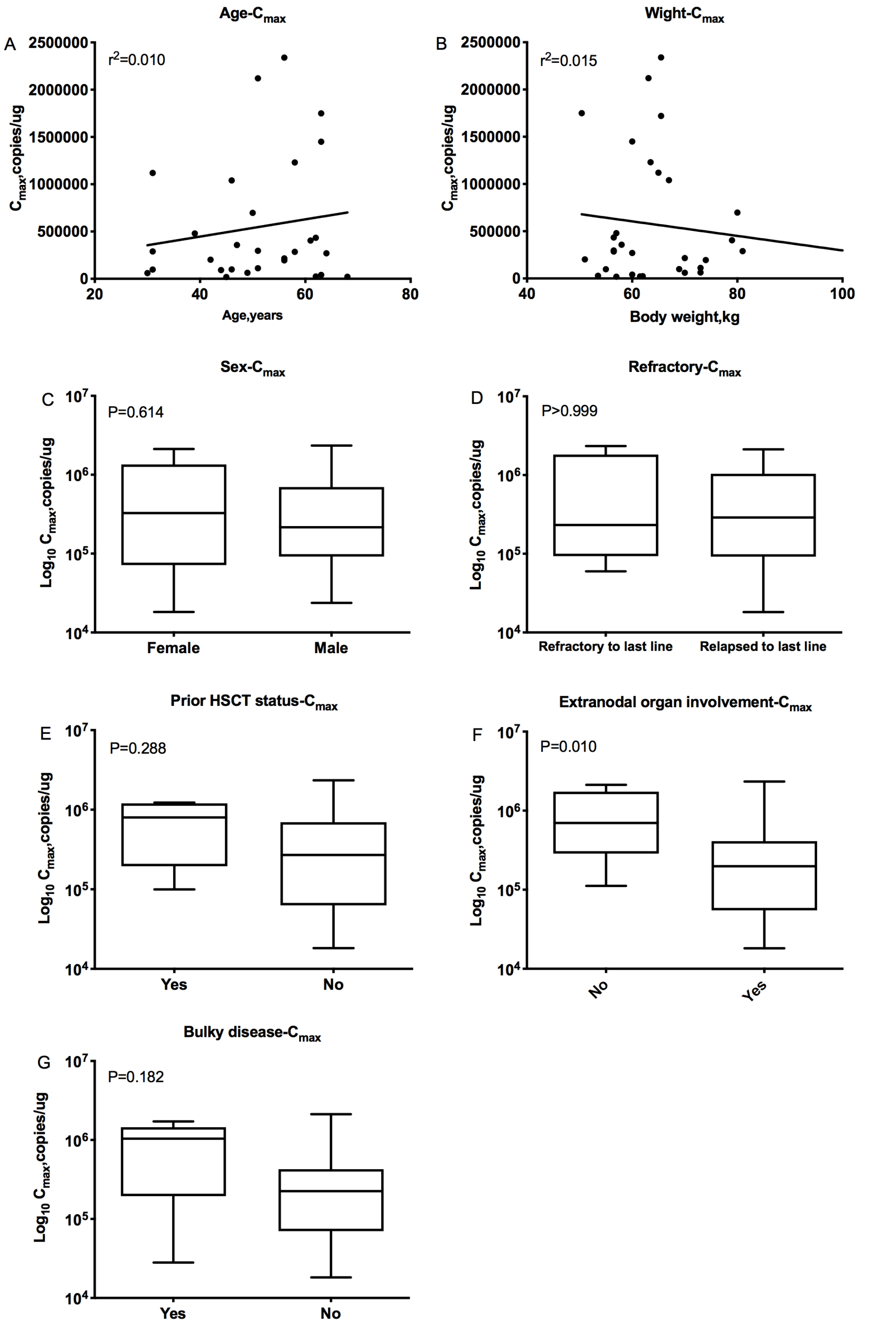

Supplementary Figure 4. Relationship between patient's characteristics and cellular kinetics. Relationship between age (A), weight (B), sex (C), refractory disease(D), prior HSCT status(E), extranodal organ involvement(F)and Bulky disease vs C_{max} (G).

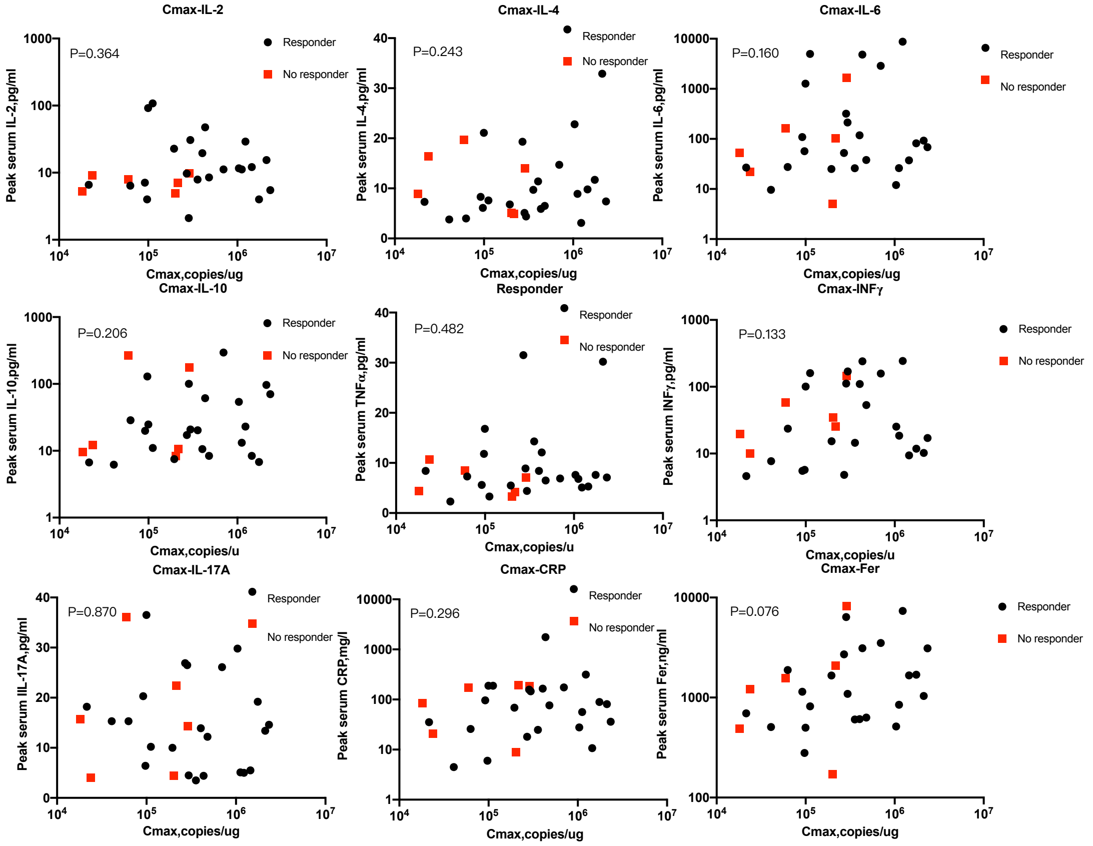

Supplementary Figure 5. Relationship between cytokines and cellular kinetics. There was no correlation between C_{max} and cytokine levels during the first 28 days.

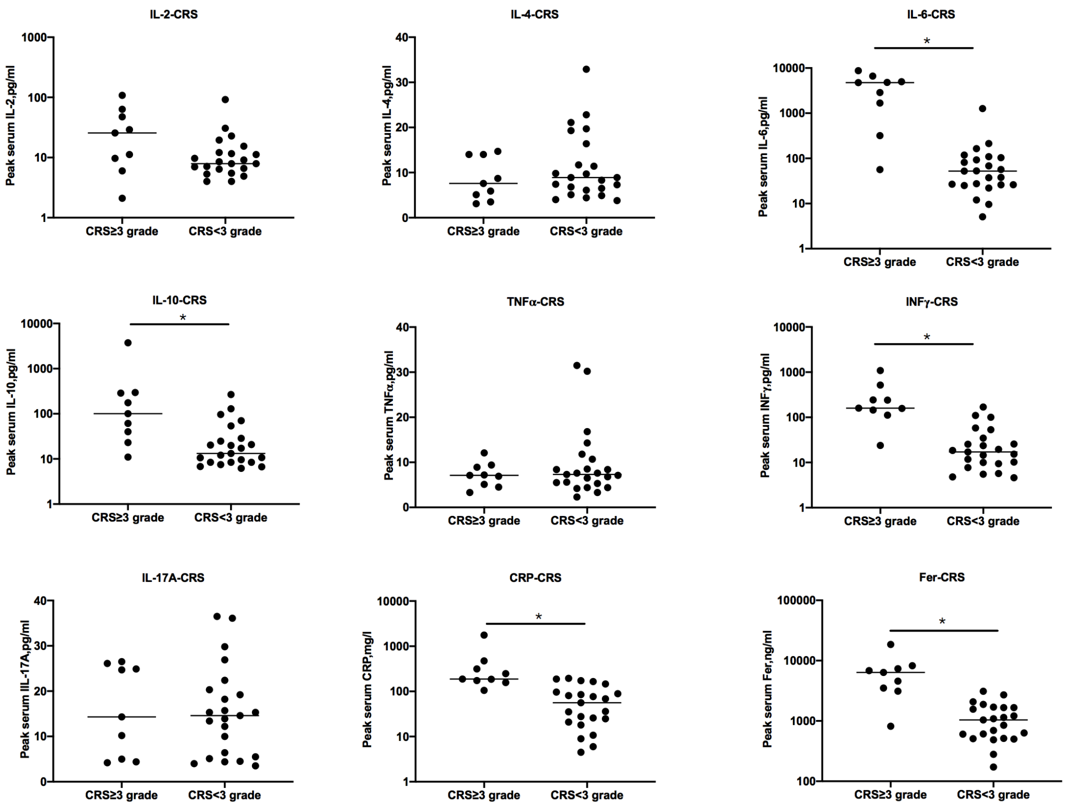

Supplementary Figure 6. Relationship between cytokines and Cytokine release syndrome(CRS). Patients with ≥3 grade CRS generally had higher levels of cytokines.
