# Supplementary material for: A Prospective Investigation of Bispecific CD19/22 CAR T Cell Therapy in Patients With Relapsed or Refractory B Cell Non-Hodgkin Lymphoma
Source: Front Oncol. 2021 May 25;11:664421. doi: 10.3389/fonc.2021.664421 (PMC8185372; doi:10.3389/fonc.2021.664421)
Supplement: Supplementary file 1 [file DataSheet_1.zip › Additional materials/Supplementary Methods.pdf]

Supplementary Methods

Eligibility criteria

(1) Histological detection confirmed non-Hodgkin lymphoma with measurable criteria

(2) Recieved more than 2 lines of chemotherapy

(3) With contraindications of hematopoietic stem cell transplantation or relapse after hematopoietic stem cell transplantation

(4) Age⩾18 and Age<78 years old

(5) Expected survival more than 3 months

(6) Karmofsky performance score ⩽ 60, and ECOG ⩾ 2

(7) Enough organ function: EF⩾50%; normal ECG; CCR⩾40ml/min; ALT and AST ⩽ 3 × upper limitation of normal, T-BIL ⩽ 2.0mg/dl; PT and APTT < 2 × upper limitation of normal; SpO2 > 92%

(8) CBC results: Hb ⩾ 80g/L, ANC > 1×10^9/L, Plt ⩾ 50×10^9/L

(9) Results of pregnant test should be negative, and agree to conception control during treatment and 1 year after CAR-T infusion

(10) Provided written informed consent before any screening procedures

Exclusion criteria

(1) Received immunosuppression treatment or steroids in recent 1 week before recruitment

(2) Uncontrolled infection

(3) HIV positive patients

(4) Active HBV or HCV infection

(5) Women in pregnancy and lactation

(6) Refuse to conception control during treatment and 1 year after CAR T infusion

(7) Uncured malignancies other than non-Hodgkin lymphoma

(8) Have participated similar trial for treating relapse/refractory non-Hodgkin lymphoma

(9) Inheritated immune deficiancy

(10) Severe heart disease
