# Supplementary material for: A Prospective Investigation of Bispecific CD19/22 CAR T Cell Therapy in Patients With Relapsed or Refractory B Cell Non-Hodgkin Lymphoma
Source: Front Oncol. 2021 May 25;11:664421. doi: 10.3389/fonc.2021.664421 (PMC8185372; doi:10.3389/fonc.2021.664421)
Supplement: Supplementary file 1 [file DataSheet_1.zip › Additional materials/Supplementary Table1.pdf]

Supplementary Table1. Multivariate Analaysis of Impact of Expansion(C_{max}), Dose, and Tumor Burden on Probability of Grad ≥3 CRS and ICANS

	Grade ≥3 CRS		Grade ≥3 ICANS	
	Parameter estimate	P value	Parameter estimate	P value
Log(Dose)	2.310	0.330	11.035	0.465
Log(C_{max})	13.335	0.152	54326.262	0.256
Tumor burden	1.032	0.027	1.098	0.227

C_{max}: maximal expansion of transgene T-cell levels in peripheral blood post infusion; CRS: cytokine release syndrome; ICANS: immune effector cell-associated neurotoxicity syndrome.
