# Supplementary figures and images for: A Prospective Investigation of Bispecific CD19/22 CAR T Cell Therapy in Patients With Relapsed or Refractory B Cell Non-Hodgkin Lymphoma
Source: Front Oncol. 2021 May 25;11:664421. doi: 10.3389/fonc.2021.664421 (PMC8185372; doi:10.3389/fonc.2021.664421)

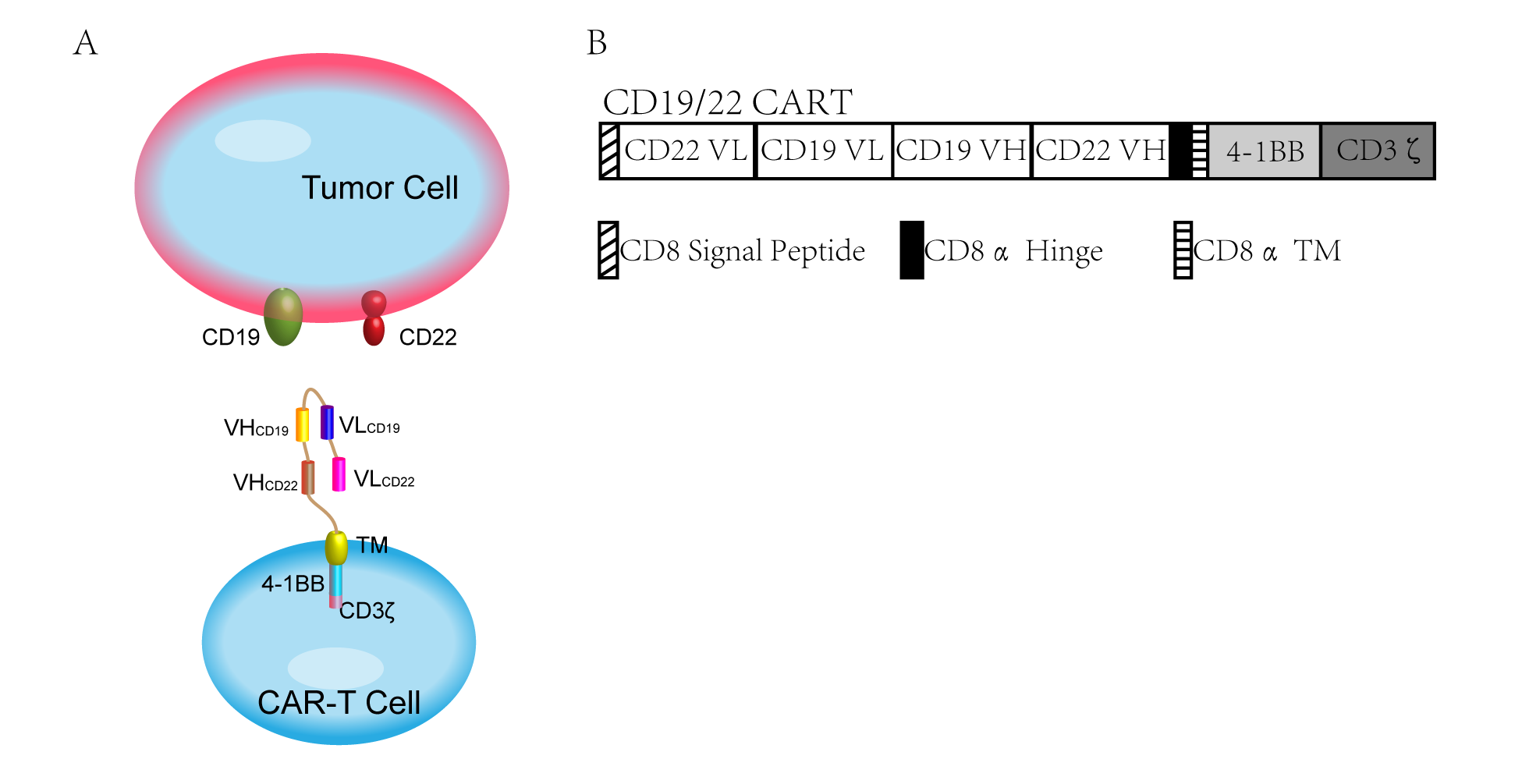

Supplement: Supplementary file 1 [file DataSheet_1.zip › Additional materials/Additional Figure 1.tif]

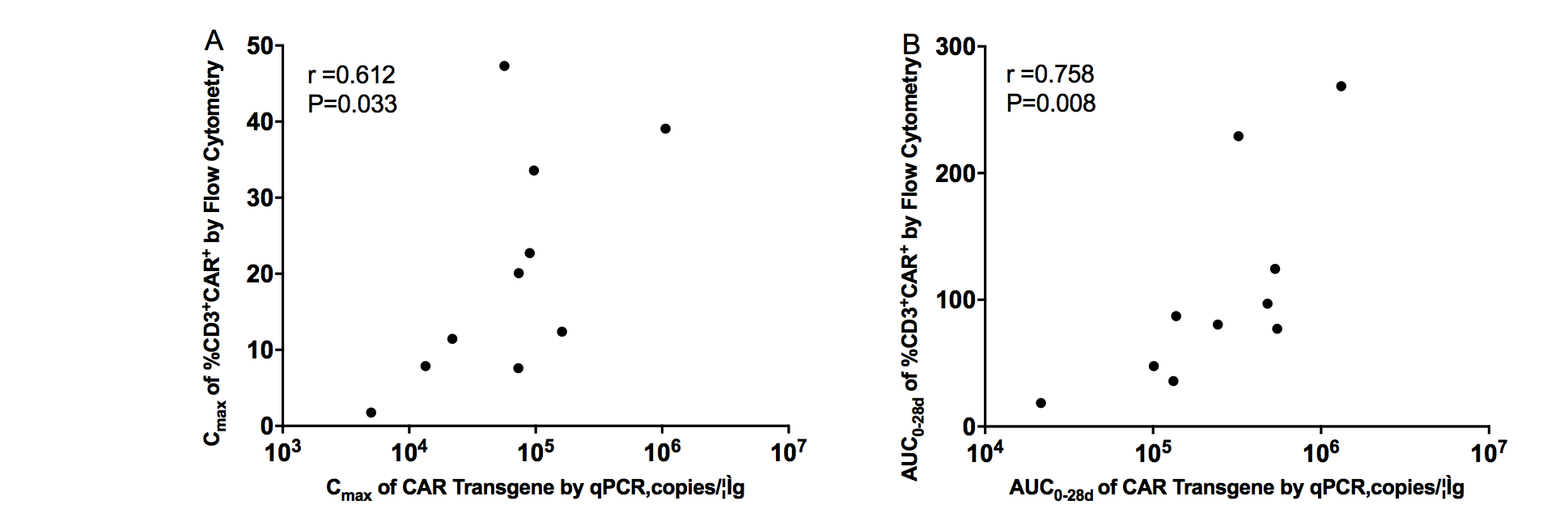

Supplement: Supplementary file 1 [file DataSheet_1.zip › Additional materials/Additional Figure 2.tiff]

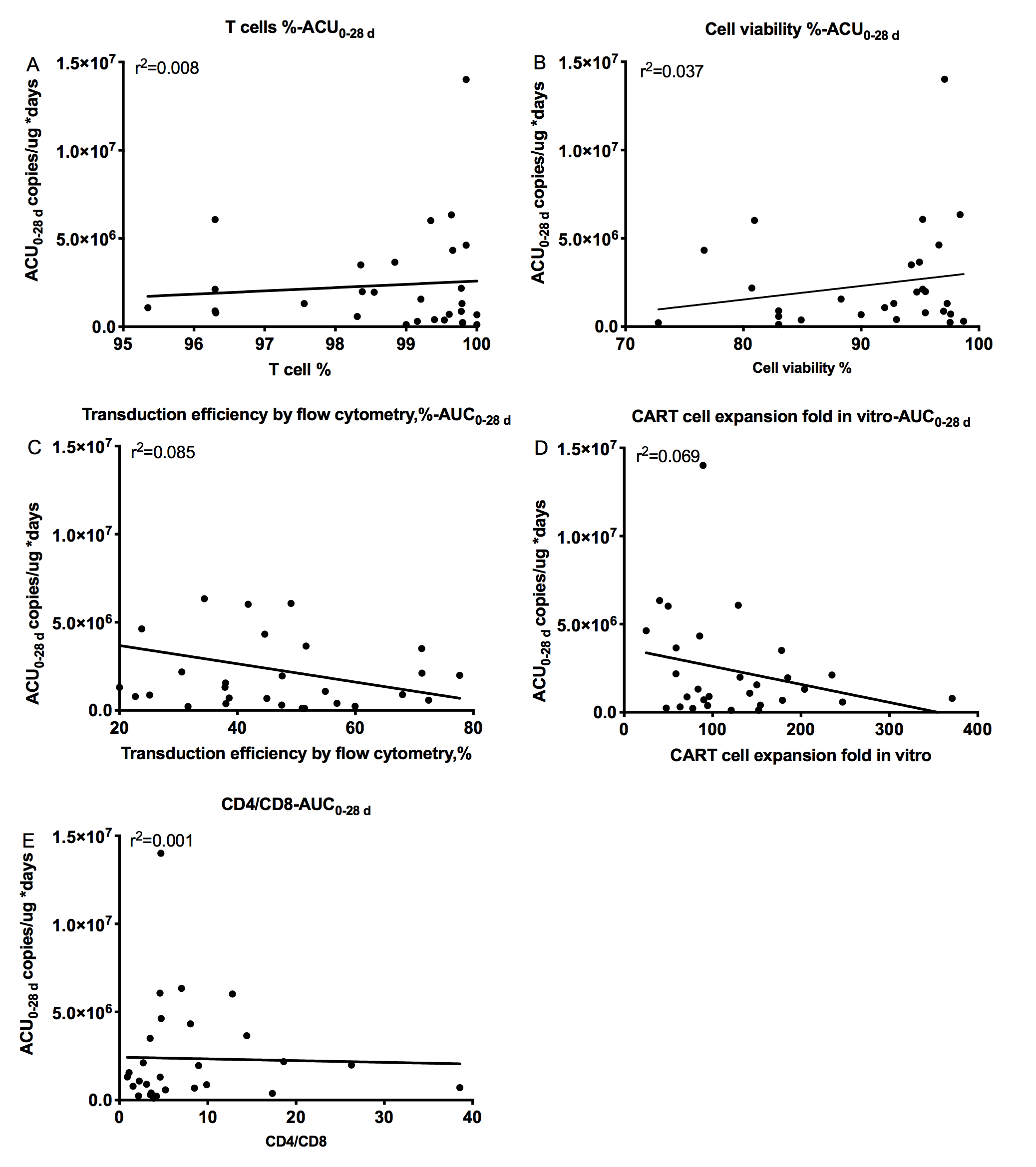

Supplement: Supplementary file 1 [file DataSheet_1.zip › Additional materials/Additional Figure 3.tiff]

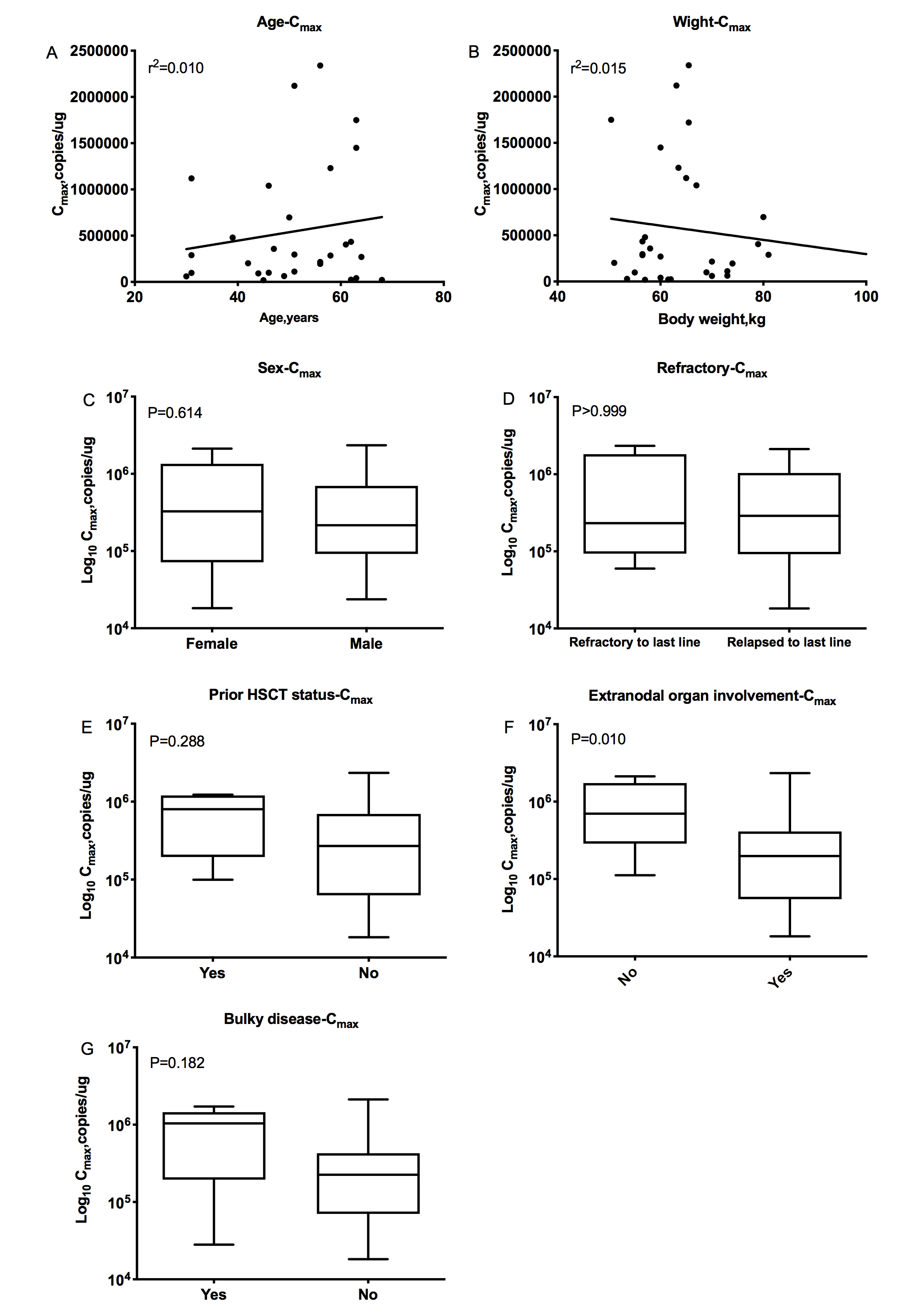

Supplement: Supplementary file 1 [file DataSheet_1.zip › Additional materials/Additional Figure 4.tiff]

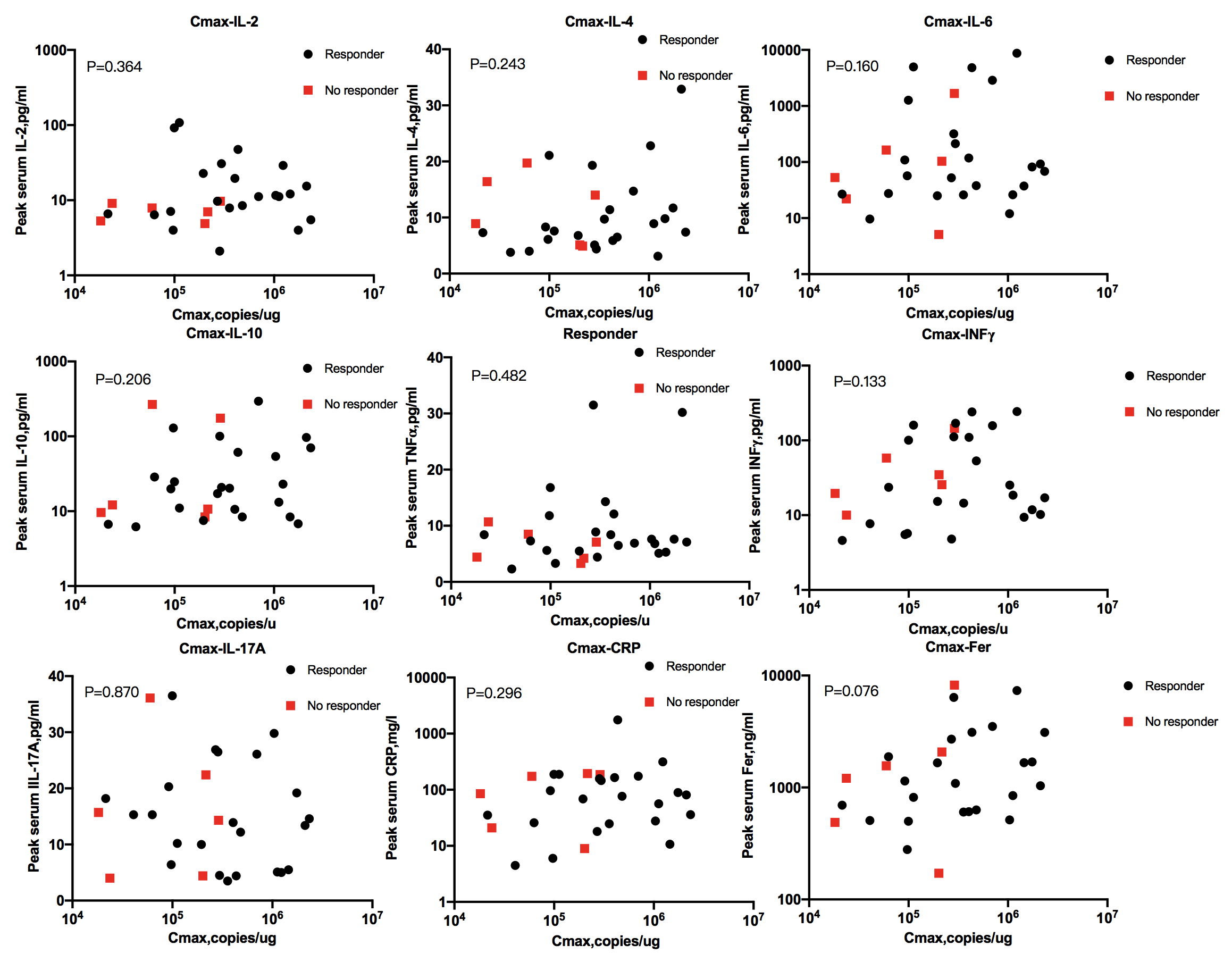

Supplement: Supplementary file 1 [file DataSheet_1.zip › Additional materials/Additional Figure 5.tiff]

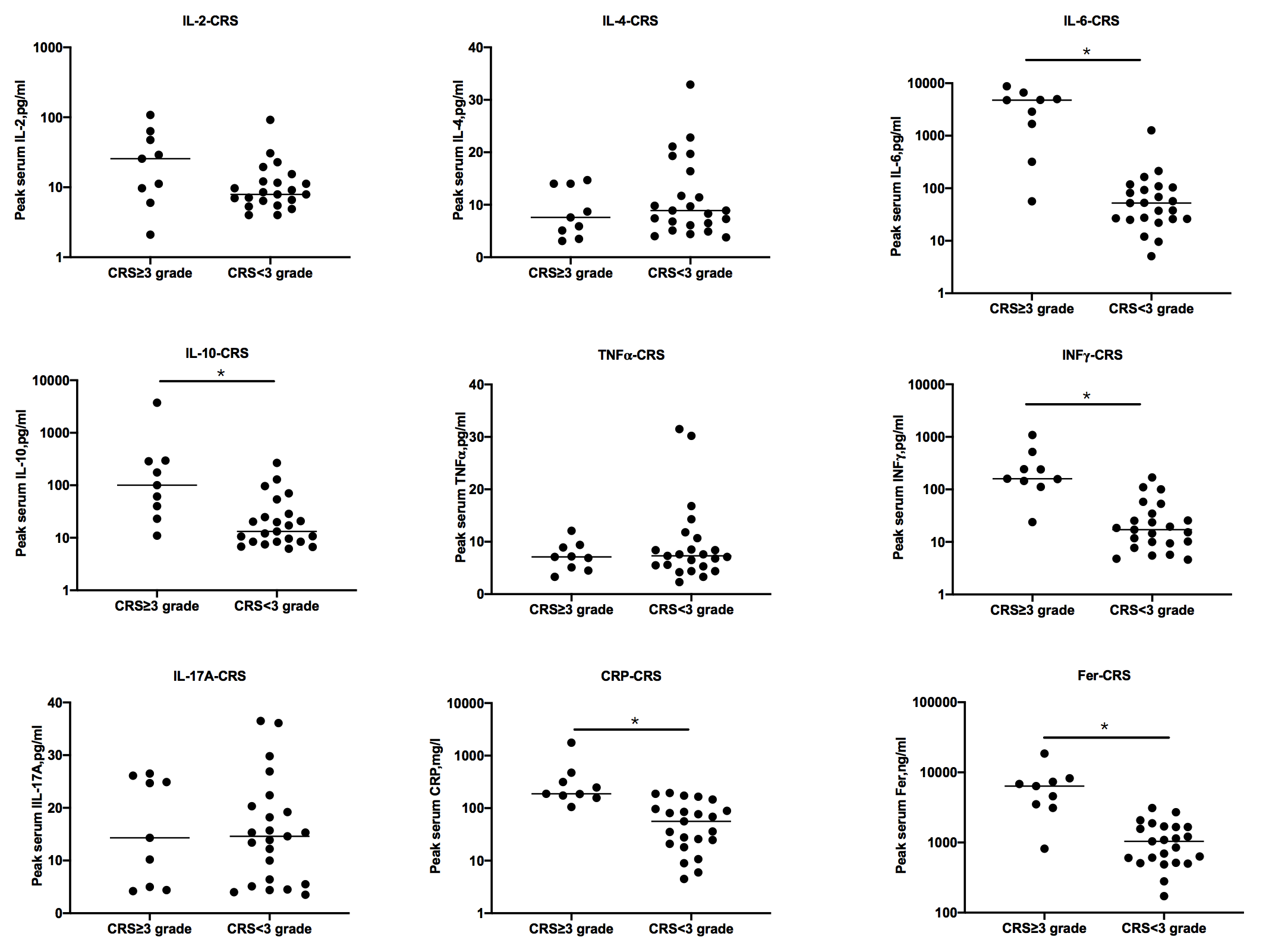

Supplement: Supplementary file 1 [file DataSheet_1.zip › Additional materials/Additional Figure 6.tiff]
